# Study of vitamin D status and vitamin D receptor polymorphisms in a cohort of Italian patients with juvenile idiopathic arthritis

**DOI:** 10.1038/s41598-020-74861-9

**Published:** 2020-10-16

**Authors:** Francesca Marini, Fernanda Falcini, Stefano Stagi, Sergio Fabbri, Simone Ciuffi, Donato Rigante, Marco Matucci Cerinic, Maria Luisa Brandi

**Affiliations:** 1 Department of Experimental and Clinical Biomedical Sciences, University of Florence, Largo Palagi 1, 50139 Florence, Italy; 2 Department of Geriatric Medicine, Division of Rheumatology AOUC, Florence, Italy; 3 Health’ Sciences Department, University of Florence, Anna Meyer Children’s University Hospital, Florence, Italy; 4 Institute of Pediatrics, Università Cattolica Sacro Cuore, Rome, Italy; 5 Department of Life Sciences and Public Health, Fondazione Policlinico Universitario “A. Gemelli” IRCCS, Rome, Italy; 6 Department of Experimental and Clinical Medicine, University of Florence, Florence, Italy

**Keywords:** Genetics, Rheumatology

## Abstract

Juvenile idiopathic arthritis (JIA) is the most common chronic arthritis of children and adolescents. Autoimmune mechanisms are suspected to have a central role in its development. Vitamin D is an immuno-modulator in a variety of conditions, including autoimmune diseases. Low levels of vitamin D have commonly been found in JIA patients, but the influence of this hormone insufficiency in JIA pathogenesis is still unclear. Vitamin D receptor (VDR) mediates a great majority of vitamin D biological activities; specific polymorphisms of the *VDR* gene have been associated with different biologic responses to vitamin D. In this study, we analysed clinical characteristics of a cohort of 103 Italian JIA patients. The distribution of *VDR* polymorphisms in affected patients versus healthy controls was evaluated, as well as if and how these polymorphic variants associate with different disease presentations (active disease vs non-active disease), different JIA subtypes, serum levels of 25-hydroxy-vitamin D and parathyroid hormone (PTH), and lumbar spine Z-score values (osteopenia vs normal bone mineral density). A great majority of our JIA patients (84.5%) showed a suboptimal vitamin D status, in many cases (84.1%) not solved by vitamin D supplementation. Vitamin D status resulted to be independent of *VDR* genotypes. *ApaI* genotypes showed a highly significant different distribution between JIA patients and unaffected controls, with both the TT genotype and the T allele significantly more frequent in patient group.

## Introduction

Juvenile idiopathic arthritis (JIA) is the most common chronic arthritis of children and adolescents, with an incidence of about 1/1000 people. About 10,000 children and adolescents are estimated to be affected in Italy.

JIA is defined as a non-infective, autoimmune, inflammatory joint disease, manifesting before the age of 16 and lasting more than 6 weeks, characterised by persistent joint pain, swelling and stiffness. In many cases, JIA undergoes a spontaneous remission after a clinical course variable from a few to several years (inactive disease), even if alternating periods of relapse and remission are common, and patients have to be constantly followed up until adulthood.

Causes of the disease are still unclear. The most accepted theory supports the influence of immunogenic mechanisms secondary to various genetic and environmental factors. Infections, in association with stress and trauma, are currently suspected to be the most responsible aetiological agents for JIA^1^.

Vitamin D is a pleiotropic hormone, exerting numerous important biological functions. Its role in the immune system has recently been recognised, as well as the fact that a persistent deficiency of its serum level and/or biological activity can contribute to the development of autoimmune diseases^2–4^.

Biological activities of vitamin D are mainly mediated by its binding to the vitamin D receptor (VDR); even a small modification in VDR activity or ligand affinity may significantly alter correct functions of vitamin D. Common polymorphic variants of the *VDR* gene may be associated with variable expression/functionality of the receptor, thus being responsible for different biological responses to vitamin D.

VDR is expressed in various immune cell lineages, such as monocytes, dendritic cells, and activated T cells. A deficiency of the active form of vitamin D has been associated with a higher risk of development of important autoimmune diseases, such as multiple sclerosis^5^, type 1 diabetes mellitus^6,7^ and systemic lupus erythematosus^8^. Low serum levels of active vitamin D have been associated with an increase of pro-inflammatory mediators and a manifestation of more active autoimmune diseases. Suboptimal vitamin D status has been reported in children with JIA^9,10^. However, the influence of vitamin D level in JIA development and disease activity is still unclear. Investigating polymorphic variants that could modulate vitamin D activity in JIA patients could help clarify whether they influence the occurrence, activity and clinical characteristics of this disease, and/or identify potential risk biomarkers.

In this study, we analysed clinical characteristics of a cohort of 103 Italian children, adolescents and young adults affected by JIA, evaluated the distribution of *VDR* polymorphisms in these JIA patients versus healthy controls, and studied whether *VDR* polymorphic variants associate with different disease presentations (active disease vs non-active disease), different JIA subtypes, serum levels of 25-hydroxy-vitamin D and parathyroid hormone (PTH), and lumbar spine Z-score values (osteopenia vs normal bone mineral density).

## Results

Our patients presented a female/male ratio of 3.90.

Forty-six patients had oligoarticular JIA (44.7%), 8 extended oligoarticular JIA (7.8%), 33 polyarticular JIA (32.0%), 7 psoriatic arthritis (6.8%), 6 ERA (5.8%); for 3 patients the specific JIA subtype was not indicated. Active disease manifested in 46 patients (44.7%), while 57 patients had a non-active JIA (55.3%).

Of 103 patients, 4 presented with vitamin D deficiency (3.9%), 83 with vitamin D insufficiency (80.6%) and only 15 showed sufficient levels of 25(OH) vitamin D (14.6%) (Table 1).

**Table 1:** Vitamin D status in JIA patients.

	Number of patients	Patients with active JIA	Patients with non-active JIA	Patients not receiving vitamin D supplementation	Patients not receiving vitamin D supplementation with active JIA	Patients not receiving vitamin D supplementation with non-active JIA	Patients receiving vitamin D supplementation	Patients receiving vitamin D supplementation with active JIA	Patients receiving vitamin D supplementation with non-active JIA
Vitamin D deficiency	4	2	2	1	0	1	3	2	1
Vitamin D insufficiency	83	38	45	33	15	18	50	23	27
Vitamin D sufficiency	15	6	9	6	3	3	9	3	6
Vitamin D high level (> 100 ng/mL)	1	0	1	0	0	0	1	0	1
Total	103	46	57	40	18	22	63	28	35

No significant difference was found in vitamin D status distribution between patients with active JIA vs patients with non-active disease.

At the time of clinical data collection, 63 patients (61.2%) were treated with vitamin D supplementation: 9 of them presented sufficient vitamin D levels (14.3%), 50 with vitamin D insufficiency (79.4%), 3 with vitamin D deficiency (4.7%), and one (1.6%) patient presented a very high level of vitamin D (190.0 ng/ml) consequent to a supplementation of 100,000 UI of cholecalciferol every 3 weeks (Table 1). No significant difference was found in vitamin D status distribution between untreated JIA patients and patients receiving the vitamin D supplementation or between the four combinations of untreated/active disease, untreated/non-active disease, treated/active disease and treated/non-active disease.

Six patients presented low levels of PTH (less than 15 pg/ml); three of them in association with vitamin D insufficiency [less than 30 ng/ml of serum 25(OH) vitamin D], two associated with normal serum values of 25(OH) vitamin D (38.5 and 34.0 ng/ml), and one was the above-mentioned patient presenting a high level of vitamin D (190.0 ng/ml).

All patients showed normal serum calcium values.

Of 102 patients, 29 showed osteopenia (28.4%), while 73 had normal BMD values (71.6%). No cases of osteoporosis were reported in our population.

The general distribution of *VDR* polymorphisms in our patients is reported in Table 2.

**Table 2:** Distribution of VDR polymorphisms in JIA patients.

*VDR* polymorphisms	*ApaI*	*BsmI*	*Cdx2*	*FokI*	*TaqI*
Genotypes	TT 53 (51.46%)	GG 31 (30.10%)	GG 60 (58.25%)	CC 46 (44.66%)	TT 35 (33.98%)
	TG 37 (35.92%)	GA 56 (54.37%)	GA 37 (35.92%)	CT 44 (42.72%)	TC 45 (43.69%)
	GG 13 (12.62%)	AA 16 (15.53%)	AA 6 (5.83%)	TT 13 (12.62%)	CC 23 (22.33%)

The distribution of genotypes of these polymorphisms was in agreement with the Hardy–Weinberg equilibrium, in both JIA patients and the control population.

Statistical comparison of the three genotypes of the *ApaI* polymorphic variant evidenced significant differences between JIA patients and control population: the TT genotype was significantly more frequent (51.46% vs 26.38%; p < 0.0001; OR 2.96) in the JIA group, while the GG (12.62% vs 26.52%; p = 0.0024; OR 0.40) and the GT (35.92% vs 47.10%; p = 0.026; OR 0.63) genotypes were both significantly less represented in JIA patients than in non-affected controls. The T allele was significantly more frequent in JIA patients than in controls (69.42% vs 49.93%; p < 0.0001; OR 2.28).

No significant differences were found for the distribution of *BsmI, Cdx2, FokI* and *TaqI* genotypes between JIA patients and controls.

The distribution of *VDR* polymorphisms was also calculated in association with JIA subtypes, clinical presentation of the disease, serum level of 25(OH) vitamin D and PTH, and bone status (Table 3).

**Table 3:** Main clinical characteristics of JIA population in relation to *VDR* polymorphisms.

	Number of patients	*ApaI*	*BsmI*	*Cdx2*	*FokI*	*TaqI*
**JIA subtype**						
Oligoarticular JIA	46	TT 27 (58.7%)	GG 11 (23.9%)	GG 26 (56.5%)	CC 22 (47.8%)	TT 11 (23.9%)
		TG 17 (37.0%)	GA 28 (60.9%)	GA 17 (37.0%)	CT 17 (37.0%)	TC 25 (54.3%)
		GG 2 (4.3%)	AA 7 (15.2%)	AA 3 (6.5%)	TT 7 (15.2%)	CC 10 (21.8%)
Extended oligoarticular JIA	8	TT 2 (25.0%)	GG 3 (37.5%)	GG 5 (62.5%)	CC 5 (62.5%)	TT 2 (25.0%)
		TG 5 (62.5%)	GA 4 (50.0%)	GA 3 (37.5%)	CT 2 (25.0%)	TC 4 (50.0%)
		GG 1 (12.5%)	AA 1 (12.5%)	AA 0 (0%)	TT 1 (12.5%)	CC 2 (25.0%)
Polyarticular JIA	33	TT 16 (48.5%)	GG 9 (27.2%)	GG 19 (57.6%)	CC 16 (48.5%)	TT 16 (48.5%)
		TG 10 (30.3%)	GA 19 (57.6%)	GA 13 (39.4%)	CT 15 (45.4%)	TC 11 (33.3%)
		GG 7 (21.2%)	AA 5 (15.2%)	AA 1 (3.0%)	TT 2 (6.1%)	CC 6 (18.2%)
Psoriatic arthritis	7 (4 of them oligo, 1 poly and 2 not indicated)	TT 4 (57.1%)	GG 5 (71.4%)	GG 4 (57.1%)	CC 1 (14.3%)	TT 2 (28.6%)
		TG 1 (14.3%)	GA 1 (14.3%)	GA 2 (28.6%)	CT 4 (57.1%)	TC 2 (28.6%)
		GG 2 (28.6%)	AA 1 (14.3%)	AA 1 (14.3%)	TT 2 (28.6%)	CC 3 (42.8%)
Enthesitis-related arthritis (ERA)	6	TT 3 (50.0%)	GG 2 (33.3%)	GG 4 (66.7%)	CC 2 (33.3%)	TT 2 (33.3%)
		TG 2 (33.3%)	GA 2 (33.3%)	GA 2 (33.3%)	CT 3 (50%)	TC 2 (33.3%)
		GG 1 (16.7%)	AA 2 (33.3%)	AA 0 (0%)	TT 1 (16.7%)	CC 2 (33.3%)
Patients for whom the specific JIA subtype has not been reported in the clinical database	3	TT 1 (33.3%)	GG 1 (33.3%)	GG 2 (66.7%)	CC 0 (0%)	TT 2 (66.7%)
		TG 2 (66.7%)	GA 2 (66.7%)	GA 0 (0%)	CT 3 (100%)	TC 1 (33.3%)
		GG 0 (0%)	AA 0 (0%)	AA 1 (33.3%)	TT 0 (0%)	CC 0 (0%)
**JIA clinical presentation**						
Non-active disease	57	TT 29 (50.9%)	GG 15 (26.3%)	GG 33 (57.9%)	CC 27 (47.4%)	TT 20 (35.1%)
		TG 20 (35.1%)	GA 35 (61.4%)	GA 22 (38.6%)	CT 25 (43.8%)	TC 27 (47.4%)
		GG 8 (14.0%)	AA 7 (12.3%)	AA 2 (3.5%)	TT 5 (8.8%)	CC 10 (17.5%)
Active disease	46	TT 24 (52.1%)	GG 16 (34.8%)	GG 27 (58.7%)	CC 19 (41.3%)	TT 15 (32.6%)
		TG 17 (37%)	GA 21 (45.7%)	GA 15 (32.6%)	CT 19 (41.3%)	TC 18 (39.1%)
		GG 5 (10.9%)	AA 9 (19.5%)	AA 4 (8.7%)	TT 8 (17.4%)	CC 13 (28.3%)
**Serum 25(OH) vitamin D**						
Vitamin D deficiency	4	TT 2 (50%)	GG 2 (50%)	GG 2 (50%)	CC 1 (25%)	TT 1 (25%)
		TG 1 (25%)	GA 1 (25%)	GA 2 (50%)	CT 3 (75%)	TC 1 (25%)
		GG 1 (25%)	AA 1 (25%)	AA 0 (0%)	TT 0 (0%)	CC 2 (50%)
Vitamin D insufficiency	83	TT 44 (53.0%)	GG 24 (28.9%)	GG 50 (60.3%)	CC 37 (44.6%)	TT 30 (36.1%)
		TG 25 (30.1%)	GA 45 (54.2%)	GA 27 (32.5%)	CT 36 (43.4%)	TC 35 (42.2%)
		GG 14 (16.9%)	AA 14 (16.9%)	AA 6 (7.2%)	TT 10 (12.0%)	CC 18 (21.7%)
Vitamin D sufficiency	15	TT 6 (40.0%)	GG 4 (26.7%)	TT 3 (20.0%)	GG 7 (46.7%)	CC 7 (46.7%)
		TG 9 (60.0%)	GA 10 (66.7%)	TC 9 (60.0%)	GA 8 (53.3%)	CT 5 (33.3%)
		GG 0 (0%)	AA 1 (6.6%)	CC 3 (20.0%)	AA 0 (0%)	TT 3 (20.0%)
Vitamin D high level (> 100 ng/ml)	1	TT 0 (0%)	GG 1 (100%)	TT 0 (0%)	GG 1 (100%)	CC 1 (100%)
		TG 1 (100%)	GA 0 (0%)	TC 1 (100%)	GA 0 (0%)	CT 0 (0%)
		GG 0 (0%)	AA 0 (0%)	CC 0 (0%)	AA 0 (0%)	TT 3 (0%)
**Bone status**						
Osteopenia	29	TT 16 (55.2%)	GG 9 (31.0%)	GG 20 (69.0%)	CC 13 (44.9%)	TT 13 (44.9%)
		TG 7 (24.1%)	GA 16 (55.2%)	GA 8 (27.6%)	CT 11 (37.9%)	TC 11 (37.9%)
		GG 6 (20.7%)	AA 4 (13.8%)	AA 1 (3.4%)	TT 5 (17.2%)	CC 5 (17.2%)
Normal BMD value	73	TT 36 (49.3%)	GG 22 (30.1%)	GG 40 (54.8%)	CC 32 (43.8%)	TT 22 (30.1%)
		TG 30 (41.1%)	GA 39 (53.4%)	GA 28 (38.4%)	CT 33 (45.2%)	TC 33 (45.2%)
		GG 7 (9.6%)	AA 12 (16.4%)	AA 5 (6.8%)	TT 8 (11.0%)	CC 18 (24.7%)
**PTH serum level**						
Low PTH serum level	6	TT 1 (16.7%)	GG 2 (33.3%)	TT 2 (33.3%)	GG 3 (50.0%)	CC 2 (33.3%)
		TG 5 (83.3%)	GA 3 (50.0%)	TC 4 (66.7%)	GA 3 (50.0%)	CT 4 (66.7%)
		GG 0 (0%)	AA 1 (16.7%)	CC 0 (0%)	AA 0 (0%)	TT 0 (0%)
Normal PTH value	97	TT 52 (53.6%)	GG 29 (29.9%)	TT 33 (34.0%)	GG 57 (58.8%)	CC 44 (44.4%)
		TG 32 (33.0%)	GA 53 (54.6%)	TC 41 (42.3%)	GA 34 (35.0%)	CT 40 (41.2%)
		GG 13 (13.4%)	AA 15 (15.5%)	CC 23 (23.7%)	AA 6 (6.2%)	TT 13 (13.4%)

No significant differences were found concerning the distribution of the five *VDR* genotypes between different JIA subtypes, active vs non-active disease, and osteopenia vs normal BMD value.

No difference was also found between the three different status of serum 25(OH) vitamin D (deficiency, insufficiency and sufficiency), when they were analysed singularly. Conversely, the analysis of vitamin D deficiency + insufficiency (87 cases) vs vitamin D sufficiency (15 cases) showed that the TG genotype of the *ApaI* polymorphism was significantly more represented in JIA patients with normal serum level of 25(OH) vitamin D (60.00% vs 29.89%; p = 0.048 with Yates’ correction; OR 0.28).

The *ApaI* TG genotype was also found significantly more represented in JIA patients with low PTH serum levels (less than 15 pg/ml) than in patients with normal PTH values (83.33% vs 32.99%; p = 0.040 with Yates’ correction; OR 10.16). The other two *ApaI* genotypes showed no significant different distribution between the two PTH groups of patients, as well as the T and the G alleles of *ApaI*.

## Discussion

The active form of vitamin D is well known to be a main actor in the homeostasis of calcium and bone remodelling. Recently, it has also been recognised as a potential immunomodulatory molecule. Deficiency of vitamin D level or activity seems to have an important role in the development of autoimmune diseases^2–4^. Low vitamin D levels have been associated with increased pro-inflammatory mediators and more active immune disorders^11,12^. Immune dysfunction is key to the pathogenesis of JIA and low levels of vitamin D have been reported in children with JIA^10^. A scoping review by Finch et al.^9^, including a total of 38 studies, evaluated possible associations of the occurrence of JIA and disease activity with vitamin D level. About 84% of the analysed studies reported that a majority of children with JIA have a suboptimal value of vitamin D. Active JIA and frequent relapses have been associated with reduced vitamin D status^9^.

Our JIA population also showed a high rate of vitamin D insufficiency (80.6%), and 3.9% of patients had vitamin D deficiency, confirming the frequent suboptimal vitamin D status reported in JIA patients. Interestingly, none of our JIA patients with a suboptimal vitamin D status showed high PTH. Conversely, six patients presented low levels of PTH, three of them in association with vitamin D insufficiency. The number of patients with vitamin D deficiency (4/103) was too small, and all of them associated with normal value of PHT, to draw any conclusion.

A study by Reed et al.^13^ showed that vitamin D supplementation did not significantly change disease activity, as confirmed by the present study. We did not find any significant difference between the relative percentages of active and non-active disease in patients receiving vitamin D supplementation vs untreated patients.

A high prevalence of vitamin D insufficiency has also been reported in the general population, and all population subgroups, from the Southern Europe and Mediterranean regions, with a greater reduction of vitamin D level in infants, children and adolescents^14^. This generally reduced vitamin D status in the European population does not help to understand the real vitamin D need in children with JIA, nor the exact role of this hormone deficiency in disease development and perpetuation. Data about 25(OH) vitamin D levels were not available for the specific sample of Italian general population we used, in this study, as control of *VDR* polymorphism distribution.

Reduced vitamin D activity could be ascribed to a reduced serum level of hormone, caused by nutritional, environmental and biological factors, or a reduced response to active vitamin D, mediated by the VDR receptor, due to genetic factors. Out of the classical activation pathway, other non-classical activation pathways of vitamin D and lumisterol, involving various cythocromes p450 enzymes other than CYP27A1 and CYP27B1, have been recently reported^15–19^ and suspected to influence serum level of active forms of vitamin D; they could be involved in the individual predisposition to develop autoimmune and/or inflammatory diseases associated to reduced levels of vitamin D. A broader investigation of biological and genetic factors influencing vitamin D synthesis and activity in JIA patients, also in comparison with the general population, could help understand how they impact the occurrence and activity of JIA, and tailor individual vitamin D adjuvant therapy. Moreover, understanding if and how genetic variants associate with JIA could unravel novel biomarkers of disease risk. Here we focused on the analysis polymorphisms of the *VDR* gene, but analysis of functional polymorphic variations and their association with vitamin D status and JIA risk, could be extended also to genes encoding for enzymes involved in vitamin D synthesis and activation, including those acting via non-classical activation pathways^15–19^.

Numerous polymorphisms of the *VDR* gene have been identified, some of them previously associated with different biological responses to vitamin D and suspected to play a role in the predisposition and development of several disorders, such as tumours, allergy, inflammation and autoimmune diseases.

In the present study, we analysed five major polymorphisms of the *VDR* gene, *ApaI, BsmI, Cdx2, FokI* and *TaqI,* in 103 children, adolescents and young adults affected by JIA. These polymorphisms have been selected because of their known effect on the modulation of *VDR* expression and activity, and/or because they have previously been associated with different phenotypes related to vitamin D activity. The *Cdx2* polymorphism maps in a promoter region of the *VDR* gene, at a binding site for the CDX2 intestinal-specific transcription factor, and it modulates vitamin D activity; the allele G exhibits 30% less transcriptional activity than the allele A.

The nucleotide substitution of the *FokI* polymorphism leads to the loss of the first ATG translation initiation region, resulting in a 3-amino acid shorter VDR protein (FF-short VDR).

The *ApaI*, *BsmI* and *TaqI* are located close to the 3′ untranslated region (3′UTR) of *VDR,* and they are suspected to affect mRNA stability and *VDR* expression, or to be in linkage disequilibrium with a truly functional allele within the *VDR* gene or nearby in another gene.

The functional effect of *FokI* genotypes in immune cells has been studied, evidencing a greater NF-kB transcription activity in the presence of the FF genotype^20^. Nf-kB activity has been associated with immune-mediated diseases^21^. Authors^20^ have hypothesised that, in individuals with low vitamin D levels and/or other predisposing genetic factors, the FF short-VDR genotype may represent an additional trigger for development of autoimmune diseases. A study of Bašić et al.^22^, on 62 JIA patient vs 91 controls, found a significantly higher frequency of the f allele of *FokI* in JIA patients. Conversely, in our study the *FokI* genotype distribution showed no difference between JIA patients and healthy controls, or between active or non-active disease presentation, confirming data of a previous study from our research group, performed on a different population of 50 children with JIA^23^.

Here, we have shown a strong association of both the TT genotype (51.46% vs 26.38%; p < 0.0001; OR 2.96) and the T allele (69.42% vs 49.93%; p < 0.0001; OR 2.28) of *ApaI* with JIA, suggesting this variant as a possible marker of JIA risk. As far as we currently know, *ApaI* polymorphism is not functional itself. However, this polymorphic variant has been associated, alone or in association with *BsmI* and *TaqI*, which with it is in strong linkage disequilibrium, to various conditions and diseases in which the vitamin D activity is known or suspected to play a pivotal function. *ApaI* maps close to the 3′UTR region of the *VDR* gene, which is known to be responsible for mRNA stability and presents numerous polymorphic variants. Variations in the nucleotide sequence within the *VDR* 3′UTR and the adjacent regions of the gene, such as the one containing *ApaI,* could exert a valuable impact in gene expression regulation. Thus, *ApaI* could be directly responsible for modulation of *VDR* mRNA stability or be in linkage disequilibrium with one or more polymorphisms inside the 3′UTR that influence it. Alternatively, *ApaI* could be in linkage disequilibrium with another adjacent gene/locus exerting a role in JIA pathogenesis.

Kostik et al.^24^ recently investigated the role of *Cdx2* and *TaqI* polymorphisms of the *VDR* gene in 192 JIA patients regarding bone status and metabolism (measured by lumbar spine DXA and biomarkers of bone turnover, respectively) and linear growth. The A allele of the *Cdx2* polymorphism was significantly more frequent in individuals with higher calcium absorption and high linear growth and the TT genotype of *TaqI* polymorphism was associated with lower BMD. No association of different *VDR* genotypes with JIA occurrence or with the presence of an active disease were performed in the study.

A study by our research group^23^ evaluated the association of *FokI* genotypes with BMD in JIA patients; patients with the ff genotype had a lower lumbar spine BMD. In the present study, we found no significant association between different genotypes of the five analysed *VDR* polymorphisms and lumbar spine BMD, as well as with the vitamin D level.

No differences in genotype distribution have been shown between different JIA subtypes, and between the presence of an active vs a non-active disease, for the *VDR* polymorphic variants analysed.

The analysis of distribution of VDR polymorphisms with respect to different levels of 25(OH) vitamin D failed to find any significant association, when deficiency, insufficiency and sufficiency were analysed separately. Conversely, when low levels of vitamin D (deficiency + insufficiency) were compared, together, with respect to sufficient serum levels of the vitamin, the *ApaI* TG genotype resulted to be significantly higher in the JIA group with normal value of 25(OH) vitamin D. The *ApaI* TG genotype was also found to be significantly more represented in JIA patients with PTH serum levels less than 15 pg/ml, with respect to patients showing normal PTH values. Nevertheless, the low number of JIA individuals with sufficient levels of vitamin D (15/103), the very low number of JIA patients presenting low PTH levels (6/103) and the absence of patients with increased PTH, as expected in presence of suboptimal status of vitamin D, appear to be biases, which reduce the strength and effectiveness of these two statistical associations. We can only speculate that these can be only two accidental associations, since neither the opposite homozygote *ApaI* genotypes (TT and GG), nor the *ApaI* opposite T and G alleles, correlated with lower vs normal level of 25(OH) vitamin D and PTH serum levels.

## Conclusions

It is suspected that a reduction of vitamin D level/activity favours the development of JIA and the maintenance of the active status of the disease. To date, only three studies^22–24^ have analysed *VDR* polymorphisms in JIA patients, principally in relation to bone status^23,24^.

This is the first study that focused on *VDR* variants in relation to the main clinical aspects of JIA. The possibility that genetic variations in the *VDR* gene could be biological markers of JIA risk is interesting from the perspective of a personalised preventive/therapeutic approach to this disease.

Insufficient and incomplete studies available, at the moment, make it difficult to confirm or confute our results.

Data need to be confirmed by studying broader JIA populations, enlarging the pathological subgroups and examining the clinical aspects of the disease and the individual response to vitamin D supplementation in detail.

Moreover, since the distribution of some of the *VDR* polymorphisms showed distinct patterns between different ethnicities, specific studies on different populations are strongly suggested, to identify possible population-specific biomarkers.

## Methods

### Patients

All experimental protocols were approved by the Ethical Review Board of the Azienda Ospedaliera-Universitaria Careggi. All patients (or legal guardians for minor patients) gave their informed consent prior to be included in this study. All clinical and laboratory procedures were performed in accordance with the Helsinki Declaration of 1975, and the 1983 revision of the same. Data were all anonymously collected and analysed, and published as aggregates.

This study included 103 Italian children, adolescents and young adults affected by JIA (82 females and 21 males; mean age at time of study recruitment 20.21 ± 7.11 years), collected from 2013 to 2015 by three Italian paediatric rheumatology centres.

According to the JIA subtypes, our patients were classified as: (1) oligoarticular JIA (affecting less than 4 joints), (2) extended oligoarticular JIA (initially affecting less than 4 joints and then extending to more than 4 joints after the first 6 months of life), (3) polyarticular JIA (affecting over 4 joints by the first 6 months of life), (4) psoriatic arthritis (simultaneous presence of JIA and psoriasis), (5) enthesitis-related arthritis (ERA; simultaneous presence of arthritis and enthesitis).

Patients were classified as inactive JIA if, at the time of study recruitment, they were in remission (i.e. no joint with active arthritis, no uveitis, normal values of inflammation biomarkers) for at least 6 months during therapy and 12 months after the suspension of therapy.

Biochemical data were collected regarding serum levels of calcium, phosphorus, PTH and 25(OH) vitamin D. According to these values, patients were classified as follows:

1. Calcium: hypocalcemia (less than 8.4 mg/dL), normal calcemia (8.4–10.4 mg/dL) or hypercalcemia (over 10.4 mg/dL)
2. Phosphorus: hypophosphatemia (less than 2.5 mg/dL), normal phosphatemia (2.5–4.5 mg/dL) or hyperphosphatemia (over 4.5 mg/dL)
3. PTH: hypoparathyroidism (less than 15 pg/mL), normal PTH value (15–88 pg/mL) or hyperparathyroidism (over 88 pg/mL)
4. 25(OH)D: deficiency (0–10.0 ng/mL), insufficiency (10.1–30.0 ng/mL) or sufficiency (30.1–100.0 ng/mL).

Bone mineral density (BMD) at lumbar spine (L1–L4) was measured as Z-score in all but one patient. Patients were classified as normal BMD (Z-score > − 1), osteopenia (Z-score between − 1 and − 2.5), or osteoporosis (Z-score < 2.5).

### Screening for VDR polymorphisms

A blood sample was collected from each patient for the extraction of genomic DNA and the performance of the genetic analysis of selected *VDR* polymorphisms. We analysed the *ApaI* (rs7975232; G > T) and *BsmI* (rs1544410; G > A) polymorphisms in intron 8, the *TaqI* (rs731236; T > C) polymorphism in exon 9, the *FokI* (rs2228570; T > C) polymorphism in exon 2, and the *Cdx2* (rs11568820; G > A) polymorphism in the promoter region of the *VDR* gene.

*Cdx2* was analysed by PCR-based Sanger sequencing, while *ApaI, BsmI, FokI* and *TaqI* were screened by PCR-based enzymatic digestions with the homonymous restriction enzymes.

A population sample of 2221 Italian unrelated individuals, without JIA, was used as control for the distribution of the genotypes of *VDR* polymorphic variants in the Italian general population.

### Statistical analyses

The allelic and genotype frequencies of all five *VDR* polymorphisms were investigated regarding their agreement with the Hardy–Weinberg equilibrium, by using the chi-squared test, in JIA and control populations.

Differences in distribution of *VDR* polymorphisms between JIA patients and non-affected controls, as well as between active and non-active disease status, different JIA subtypes, different serum levels of 25(OH) vitamin D and PTH, and different lumbar spine Z-score values were statistically evaluated by chi-squared test. The Yates’ correction was used with groups of less than twenty individuals. Statistical significance was considered with a p value less than 0.05.

## Data Availability

The dataset used and analysed during the current study is available from the corresponding author on reasonable request.
